# Evaluating the influence of persuasive systems design on continuance intention, perceived effectiveness, and weight loss

**DOI:** 10.1177/20552076261461365

**Published:** 2026-06-16

**Authors:** Sharon Nabwire, Eunice Agyei, Heta Merikallio, Markku J. Savolainen, Janne Hukkanen, Harri Oinas-Kukkonen

**Affiliations:** 1 Faculty of Information Technology and Electrical Engineering, 6370 University of Oulu, Oulu, Finland; 2 Research Unit of Biomedicine and Internal Medicine, 6370 University of Oulu, Oulu, Finland; 3 Medical Research Center Oulu, 6370 Oulu University Hospital and University of Oulu, Oulu, Finland; 4 Department of Medicine, Oulu University Hospital, Oulu, Finland

**Keywords:** digital health interventions, persuasive systems design, behavior change support systems, structural equation modelling, weight management

## Abstract

**Background:**

Digital health interventions for behavior change show great promise in supporting patients who need to self-manage or prevent chronic diseases. Persuasive features are crucial to the continued use of behavior change support interventions, though less is known about how users’ perceptions of these features shape evaluations of technology acceptance and health outcomes.

**Objective:**

This study examined how perceived persuasive design principles influence perceived persuasiveness and technology acceptance factors (perceived effort, continuance intention, perceived effectiveness) in a mobile digital health intervention to support weight management.

**Methods:**

The study is a secondary analysis of a subset of the data (n=181) from a randomized controlled trial of users of a weight-focused mobile application. Data were analyzed using PLS-SEM to test the proposed model and examine the relationships among the study variables.

**Results:**

Our findings showed that persuasive design features of the primary task, dialogue support, credibility support, and postulates of unobtrusiveness and transparency explained 65.6% of the variance in perceived persuasiveness. In turn, perceived persuasiveness influenced perceived effort, continuance intention, and perceived effectiveness. The model explained 12.7% of the variance in weight change, with medium predictive power. Moreover, introducing age as a moderator between perceived effectiveness and weight change increased the model’s explanatory power (from R^2^ =12.7% to 16.3%).

**Conclusion:**

The findings contribute to the theoretical understanding of persuasive systems design and technology adoption in digital health interventions, offering insights for researchers and designers on how these principles can support health behavior change.

## Introduction

Obesity and overweight-related challenges are on the rise.^1,2^ However, these conditions can often be prevented or mitigated through proactive management of health that advocates healthy behaviours and lifestyle choices.^3^ One way to achieve this is through the development of digital health interventions that support behavior change. Recent evidence highlights the effectiveness of such approaches; for instance, a meta-analysis of randomized controlled trials found that smartphone app-based interventions significantly reduce body weight, BMI, waist circumference, and fat mass in adults with overweight and obesity.^4,5^ The analysis also emphasizes the need to incorporate structured behavior change strategies to optimize outcomes.^4,5^ Behaviour change support systems (BCSS) are information systems that persuade users to comply, change, adopt, or reinforce desired behaviours without coercion or deception.^6^ To encourage positive behavior change, BCSS may use different technologies, behavior change theories, and user-centred design concepts from different disciplines.^7^ Due to their potential to support behaviour change across several domains, including physical activity, stress reduction, and other health areas, such systems have attracted significant interest in recent years.^8–10^ They usually have educational and behavior change components,^11^ and require careful analysis and selection of appropriate features to support the desired behavioral changes via the intervention.^12^

Persuasive design principles are rooted in behavioral science and psychology and are harnessed to prompt users toward desired actions.^7^ Behaviour change may occur when motivation, ability, and triggers intersect.^13^ Also, employing the wealth of knowledge on persuasive system design (PSD) principles and behavior change techniques can facilitate the adoption of sustainable behavior change, essential for successful long-term maintenance of weight loss.^14,15^ Typical content in weight management apps includes health-related information on nutrition, diet and physical activity; self-monitoring tools for tracking weight and calories; and ways to interact with experts and other users within the app.^16^ The most prevalent PSD principles used in weight management apps include self-monitoring, personalization, tailoring, and reminders, together with behavior change theories.^17^ Previous research has shown the use of persuasive design in weight management apps. The findings of Asbjørnsen et al.^14^ identified behavior change techniques and persuasive systems design principles as effective for promoting behavior change toward weight management. Furthermore, a meta-analysis by Webb et al.^18^ showed that theory-driven interventions that support behavior are effective in improving health-related outcomes, and their findings are consistent with a meta-synthesis of meta-analyses.^19^ Both above-mentioned studies called for an investigation into the factors that make interventions effective, whether system-related or user-related.

Quantitative studies on user perceptions have used the technology acceptance model and its derivatives to examine the factors that lead users to accept, adopt, and use a system.^20–23^ Other studies considered users' perception of system-related features and their impact on the intention to continue using a system.^24,25^ Alongside quantitative approaches, qualitative studies have explored how users perceive and interact with persuasive features in digital weight management, offering insights into user experiences and design preferences.^14,26,27^ However, while these studies show that persuasive features are crucial for the continued use of a BCSS,^24,28^ less is known about how users’ perceptions of these features shape technology acceptance evaluations and how these, in turn, affect health outcomes such as weight loss. As BCSS adoption increases, clarifying this gap is necessary for understanding user experiences and the effectiveness of these systems. This study, therefore, examines how perceived PSD design principles influence perceived persuasiveness and BCSS acceptance factors of perceived effort, continuance intention, perceived effectiveness, and ultimately weight loss outcomes. A structural equation model is proposed and validated using a subset (n=181) of data from a randomized controlled trial (RCT) focused on a weight management mobile intervention.

## Theoretical background

### Persuasive systems design

The Persuasive System Design (PSD) model is a three-step model for researching, analysing, and developing systems that support behavioral change.^7^ In the first step, the model advocates understanding key assumptions that underpin information technologies: (1) they are available 24/7 and thus afford many opportunities to persuade their users, (2) people desire to have consistency between their worldviews and actions, (3) persuasion occurs gradually, (4) persuasive appeals occur via the direct route, indirect route or both, (5) systems should be both useful and easy to use, which is rooted in technology acceptance research,^20^ (6) systems should be unobtrusive, fitting to the user and their environment, and (7) systems should be transparent, disclosing the intention and any designer bias that may exist. Having established an understanding of these assumptions, the second step involves an analysis of the persuasion context, which includes determining the intended change, investigating the problem domain, user characteristics, the technology-related factors, and determining the strategy for persuasion. The strategy considers the message, how the message is presented through the direct, indirect route, or both, and possible ethical issues.^7^ The last step involves analysing and selecting suitable persuasive software features from four categories to support the user in performing the system’s primary tasks (Primary task support), interacting with the system (Dialogue support), enhancing the system’s perceived credibility (Credibility support), and facilitating social support for users who need it.^7^ The features in these categories can be translated into software functionalities.^29^ These steps informed and guided the development of the weight management intervention used in this study.

### Technology acceptance and behavior change

The technology acceptance model (TAM)^20^ is often used in information systems research to assess how people use and adopt new technologies. The acceptance of these technologies depends on their features and characteristics,^30^ which are part of persuasive systems design. For example, the fact that technology should be useful and easy to use is a fundamental principle of persuasive design to ensure a positive user experience.^7^ While usefulness is defined as “*the degree to which a person believes that using a particular system would enhance his or her job performance*”, ease of use seeks to assess “*the degree to which a person believes that using a particular system is free from physical and mental effort*”.^20^ The effort required to use a system has been described as the finite resources needed to complete tasks within it.^31^ According to research,^32^ people adopt technologies if they perceive them to be effective. Effort expectancy (ease of use) and performance expectancy (usefulness) are used to explain individuals’ behavioral intention to use a system.^33^ Furthermore, studies have incorporated elements of PSD to explore their impact on these factors and a user’s intention to continue using the system. For instance, Wiafe et al.^28^ and Lehto et al.^24^ have evaluated how persuasive features affect the intention to continue using BCSS, specifically in the contexts of academic social networking sites and weight management systems, respectively.

## Hypothesis development

**Figure 1.**
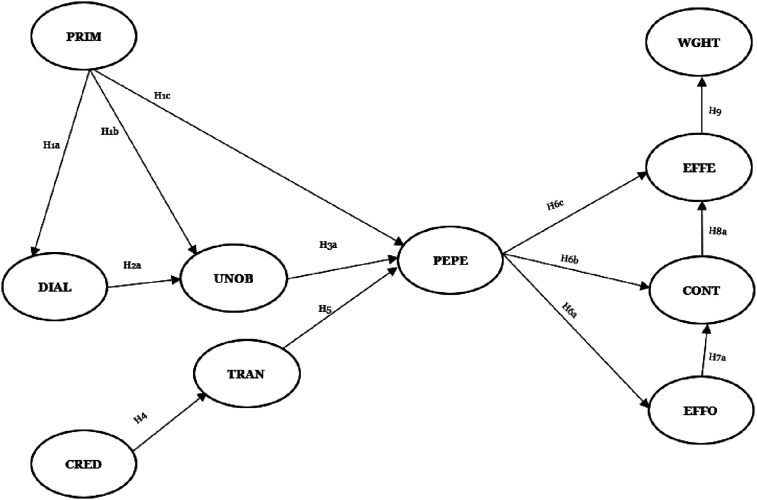
Research model. *Note.* PRIM = Primary task support; DIAL = Dialogue support; CRED = Credibility support; UNOB = Unobtrusiveness; TRAN = Transparency; EFFO = Perceived effort; CONT = Continuance intention; EFFE = Perceived effectiveness; WGHT = Weight change.

System features and characteristics affect user acceptance of technology.^30^ To this end, we formulated a model (Figure 1) to examine the persuasiveness of BCSS features (PRIM, DIAL, CRED) and characteristics (unobtrusiveness, transparency) implemented in the mobile intervention and their effect on perceived effort, continuance intention, and perceived effectiveness, ultimately influencing weight loss. Social support features were excluded from the study as they were not part of the intervention design. The model captures the functional roles of the PSD features employed in the intervention, incorporating only theoretically justified pathways rather than assuming that all PSD elements are interconnected.

### Primary task support

Primary task support (PRIM) refers to the means provided by a system to aid a user in performing his/her original core tasks via the software.^7,34,35^ It consists of features that enable users to reflect on their behavior through self-monitoring, organize complex tasks into smaller, manageable subtasks through reduction, and encourage users to practice the desired behavior through rehearsal. Facilitating these tasks requires user interaction to seamlessly integrate into their routine with little or no obtrusion, which is crucial to providing a positive user experience.^6^ If a system fails to fit flawlessly into the daily lives of its users, the likelihood of its usage can diminish and affect task performance. Besides, PRIM features promote a positive effect that can influence the system’s persuasion.^36^ We propose the following hypothesis:

H1a: Primary task support is positively related to dialogue support
H1b: Primary task support is positively related to unobtrusiveness
H1c: Primary task support is positively related to perceived persuasiveness

### Computer-human dialogue support

To support the user’s interaction with a system, prompts (under Computer-human Dialogue Support, DIAL) may be necessary to alert users to undertake tasks through recommendations, hints, or tips about the desired behavior, reminders, and positive feedback to reinforce that behavior.^7^ These features act as motivational elements, encouraging users to carry out the main tasks they intend to accomplish with the system.^37^ DIAL features emerge as the most significant aspects of the system, greatly influencing users’ perceptions and acceptance of it.^38^ These reasons led us to hypothesize that:

H2a: Dialogue support positively influences unobtrusiveness
H2b: Dialogue support mediates the relationship between primary task support and unobtrusiveness

### Unobtrusiveness

Unobtrusive design enables technologies to fit seamlessly into users’ daily routines with minimal disruption or interference.^7^ This implies that an unobtrusive system is aware of changes in the user’s life and adjusts accordingly, delivering messages (e.g., reminders, suggestions) when necessary. When such provisions are made, users can engage in the desired behavior by interacting with the system,^39^ potentially increasing the persuasiveness of the system. Hence, we hypothesize that:

H3a: Unobtrusiveness is positively related to perceived persuasiveness
H3b: Unobtrusiveness mediates the relationship between primary task support and perceived persuasiveness

### Perceived credibility support and transparency

The credibility of a system (CRED) influences its persuasiveness if it considers factors such as trustworthiness and expertise. Users are likely to be confident in using a system that they perceive as credible. While trustworthiness captures the perceived goodness, reliability, or dependability of a system,^7^ expertise considers the knowledge, experience, or competence of a system, and as such, credible systems are likely to be perceived as having high levels of both trustworthiness and expertise.^40^ Transparency as a system characteristic aims for openness,^6^ as it helps build user trust and confidence in the system.^41^ Therefore, if users have this trust in the system, they will perceive it as transparent. When evaluating health communication, source credibility is considered to be positively associated with perceived message transparency,^42^ thereby increasing the persuasive impact. While prior studies position transparency as an antecedent to credibility,^43^ we propose that credibility through trustworthiness and expertise enhances perceived transparency. We hypothesize that:

H4: Perceived credibility is positively related to transparency
H5: Transparency positively influences perceived persuasiveness

### Perceived persuasiveness

Perceived persuasiveness of a system refers to the perception of the extent to which design principles can influence behavior change.^44^ It consists of several factors, including the presence and usefulness of persuasive features for a target goal. According to Beerlage-deJong et al.,^45^ the perception of PRIM, DIAL, and CRED features, as well as unobtrusiveness, impacts the perceived persuasiveness. Perceived persuasiveness has also been defined as the capacity of a system to influence behavior change positively.^9^ The system’s ability to persuade and a favourable user experience encourage people to engage with it.^6^ Consequently, the extent to which the system is seen as persuasive affects both its usage and the intention to keep using it.^46^ Therefore, users’ positive perception of the system influences their decision to keep using it, which in turn supports behavior change.^47^ Likewise, when the system persuades people, they will perceive it as easy to use and subsequently effective in helping them achieve their intended outcomes. Hence:

H6a: Perceived persuasiveness is positively related to perceived effort
H6b: Perceived persuasiveness is positively related to the intention to continue using the system
H6c: Perceived persuasiveness is positively related to perceived effectiveness

### Perceived effort, continuance intention, and perceived effectiveness

Perceived effort, continuance intention, and perceived effectiveness are key concepts in understanding how technology is used and adopted.^33,48,49^ Effort expectancy is the degree of ease associated with technology, and it influences the behavioral intention to use a system.^33,50^ It relates to the user’s assessment of the cognitive and physical effort required to interact with the system. When users perceive the system as easy to use, they will continue using it. Besides, a system is unlikely to be highly persuasive if it is useless or challenging to use.^7^ In this study, effort was measured such that higher scores indicate lower effort requirements (e.g., “using the system does not require a lot of effort from me”; See Appendix A). We therefore propose that:

H7a: Effort is positively related to continuance intention
H7b: Effort mediates the relationship between perceived persuasiveness and continuance intention

Continuance intention refers to the aim to continue using a system.^48,49^ Continuous usage of a system may enhance its perceived efficiency or effectiveness, especially if minimal effort is required.^33,50^ When users plan to keep using a system, their continued interaction can reinforce and even increase their perception of its effectiveness.

H8a: Continuance intention is positively related to perceived effectiveness
H8b: Continuance intention mediates the relationship between perceived persuasiveness and perceived effectiveness

Perceived effectiveness assesses the system’s capacity to execute its functionality successfully. If users view the system as effectively influencing their behavior, it can lead to weight loss. Prior BCSS research indicates that continued usage of effective systems can lead to the attainment of behavioral goals such as weight management.^24^ On this logic, we hypothesize that:

H9: Perceived effectiveness is positively related to weight change

## Research methodology

This research is a secondary analysis of data from a larger RCT^15^ collected six months after participants used a mobile-based health intervention. A survey instrument was developed using theories derived from conceptual and empirical studies to assess the constructs of our model (Appendix A).^7,24,33,46,48,49^ The latent variables were measured with reflective multiple-item scales adapted from constructs operationalized in prior literature.^20,39^ The transparency measure, while not previously operationalized as a standalone measure, is firmly grounded in the established PSD framework,^7^ consistent with acceptable practice for emerging constructs in this field. This grounding in both established empirical measures and theoretical foundations demonstrates psychometric suitability. A 7-point Likert scale ranging from “Strongly agree = 1” to “Strongly disagree = 7” was used to measure the extent to which the respondents agreed with the statements regarding persuasive features, postulates, perceived persuasiveness, and the BCSS acceptance characteristics (perceived effort, continuance intention, and perceived effectiveness).

### Mobile health intervention

**Figure 2.**
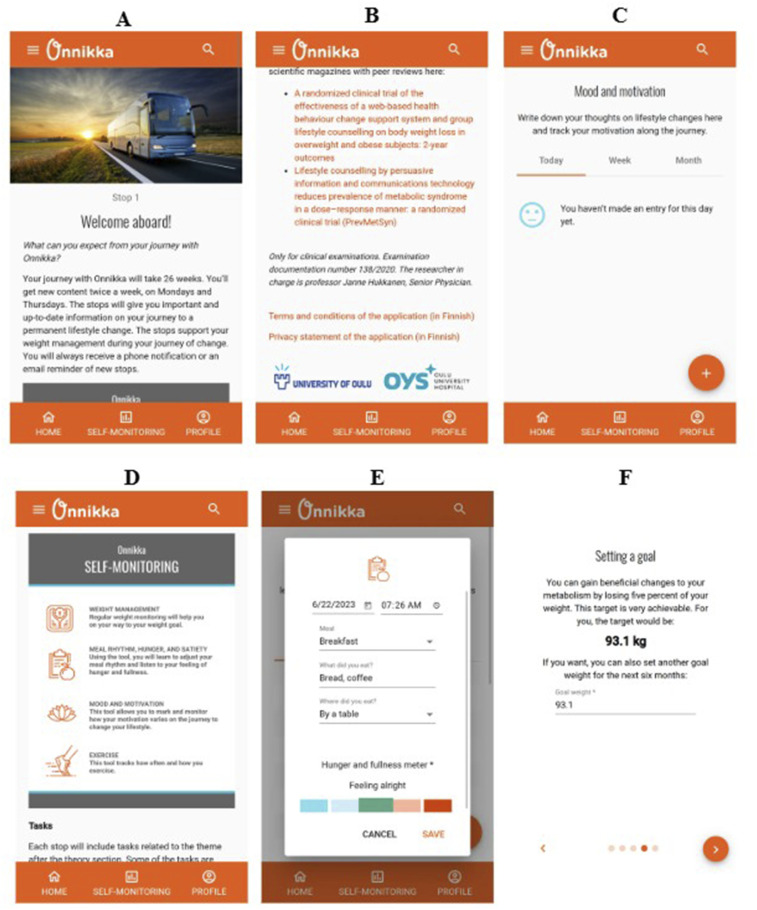
Mobile intervention screenshots.*Note.*Onboarding (A, B); Credibility support (CRED)—transparency and verifiability (B); Dialogue support (DIAL)—reminders (C); Primary Task Support (PRIM)—self-monitoring (D, E) and goal setting (F).

*Onnikka* is a digital mobile health intervention (Figure 2) designed to facilitate weight management among obese patients between the ages of 18 and 65 in an RCT. The RCT study design was approved by the Ethics Committee of the Northern Ostrobothnia Hospital District, with approval number *138/2020*, registered at ClinicalTrials.gov [identifier number: *NCT04558801*], and regulated by the Finnish Medicines Agency. In a *Finnish* local dialect, *Onnikka* translates to “a bus”. The intervention, therefore, metaphorically uses a “bus ride” journey through symbolic “stops” to illustrate the user-guided process of behavior change. Educational content, self-monitoring tasks, and reflective exercises are presented to users at the “stops”. Users received content on physical activity, motivation, behavior, and nutrition, created by health professionals, twice a week during the first six months of the intervention. In the following 6-month period, users had access to this content, and no new content was provided. Within this timeframe, some content (based on the user’s system interaction) from the previous months was provided for three weeks. The system was built from the ground up in accordance with the PSD model’s guidelines. After completing the PSD design process, persuasive software features from PRIM, DIAL, and CRED were implemented in the intervention. The PSD principles operationalized in this study, corresponding to the constructs included in the measurement model, are described below.

• For PRIM, the application enabled continuous self-monitoring by allowing users to record their body weight and behavior indicators, including dietary intake, perceived hunger, exercise, and motivation levels. After the first half of the intervention, selected content materials were tailored based on users’ responses to earlier tasks. Content delivery was structured to reduce task load (twice a week).
• Under DIAL, users received feedback through reminders and praise messages. Reminders were delivered when expected inputs were missing (e.g., missed weight recordings), while praise messages were sent in response to positive progress (e.g., achieving weight goals).
• CRED was supported through evidence-based, neutrally presented content (Trustworthiness). Expertise was demonstrated through scientifically grounded content.
• PSD postulates of unobtrusiveness and transparency were also supported. Unobtrusiveness was addressed by delivering reminders in a non-intrusive manner. Transparency was ensured through clear communication of the system’s intent, explanation of key strategies (e.g., stop-related tasks) during onboarding, disclosure of data use, and informing users of the intervention's research purpose.

### Data collection

Participants in the RCT were recruited from Oulu University Hospital, the University of Oulu, and on-campus private companies. The invitation was also open to other interested individuals, including friends and family from the aforementioned establishments. Participants were included if they satisfied the following criteria: body mass index between 30 and 40 kg/m^2^, aged 18 to 65 years, having access to a mobile phone or tablet, and not participating in other weight-loss programs during the intervention period. Participants were excluded if they had uncontrolled hypothyroidism, were receiving oral corticosteroid therapy, were pregnant or breastfeeding, had cardiovascular disease preventing physical activity, lacked sufficient *Finnish* language skills (main language of delivery), had planned or previously undergone bariatric surgery, or were using anti-obesity medications.^15^ Altogether, 200 participants were included in the parent study and randomly assigned to receive the mobile intervention immediately (Group 1, n=100) or after six months (Group 2, n=100). This sample size was calculated based on an estimated 3.2 kg difference in weight between the groups and a 6.7 standard deviation change in weight as the primary outcome. This calculation indicated that a sample size of 69 participants per group was needed. However, 100 individuals per group were estimated to provide 80% power and a 5% type 1 error, assuming a dropout rate of 30% (See details in parent RCT study^15^). The survey to elicit user perceptions of the mobile intervention was distributed to participants both online, using the software tool Webropol 2.0, and on paper.

In this study, we use a subset of the data (n = 181) comprising participants from both groups 1 and 2 who completed the survey after six months of using the intervention. A sample size justification was conducted based on statistical power considerations for PLS-SEM.^51,52^ Using a significance level of 5% and a statistical power of 80%, the minimum required sample size was estimated based on the smallest path coefficient in our model (β = 0.237), following the inverse square root method proposed by Kock and Hadaya.^52^ This method indicated a minimum required sample of 69.^52^ Since the final sample size exceeds this threshold, it is considered sufficient for the analysis. While the commonly applied 10-times rule^53^ provides a basic guideline for sample size estimation, the model’s number of constructs and paths has little effect on the sample size requirements,^51^ therefore, a power-based approach was used to justify the sample size in this study.

The outcome variable in this study is weight change. Weight measurements were taken at the hospital at baseline and at six months after using the intervention. Research shows that maintaining a five percent weight loss is clinically meaningful for improving health outcomes.^54^ Weight change was therefore categorized into four categories based on the extent of weight loss: x ≤ 0% weight loss as category four, 0% < x ≤ 2% weight loss as category three, 2% < x ≤ 5% weight loss as category two, and x > 5% weight loss as category one. This categorization, therefore, helps illustrate variations in weight loss outcomes rather than define separate outcome groups within the model.

## Analysis and results

Data from 181 participants, predominantly female (n=158), with a mean age of 47 (Appendix B), were analyzed using structural equation modelling (SEM). SEM is a statistical technique that combines elements of regression to determine and examine the relationships between observed and latent variables.^55^ While statistical analysis, such as correlation, regression, and tests of mean differences, such as ANOVA or t-tests, offer limited modelling capabilities, especially for causal modelling, SEM methods are suitable for causal modelling.^55,56^ The partial least squares structural equation modelling (PLS-SEM) method was used to explore and estimate the strength of relationships between the latent variables and determine how well the model explains the target constructs of interest.^51^ PLS-SEM can be used to estimate very complex models^51^and has relaxed data requirements that are suitable for the ordinal nature of the data collected.^57^ SmartPLS 4 was used to conduct the analysis,^58^ first by assessing the measurement model, followed by the structural model to test the hypothesized relationships, using the proposed guidelines by Hair et al.^51^

### Assessment of the measurement model

The item loadings and composite reliability scores were used to assess the internal consistency of the measurement model. Item loading scores (Appendix A) were greater than the acceptable threshold of 0.708.^51^ Additionally, Cronbach’s α and composite reliability for internal consistency were above the required threshold of 0.7.^51^ Moreover, the average variance extracted values of all the latent variables were above the recommended minimum of 0.50,^51^ thus demonstrating adequate convergent validity. See Table 1 for detailed results.

**Table 1.** Construct reliability and validity.

​	Cronbach’s α	Composite reliability (rho_c)	Average variance extracted (AVE)
PRIM	0.903	0.940	0.839
DIAL	0.753	0.888	0.799
CRED	0.972	0.980	0.924
UNOB	0.874	0.922	0.799
TRAN	0.894	0.934	0.825
PEPE	0.874	0.923	0.800
EFFO	0.835	0.900	0.751
CONT	0.800	0.881	0.712
EFFE	0.888	0.931	0.818

The discriminant validity of the model was assessed using the Heterotrait-monotrait ratio (HTMT) criterion as proposed by Henseler et al.^59^ All constructs, as shown in Table 2, met the rigorous HTMT .85 threshold except PRIM and DIAL (0.900), which fell within the more relaxed HTMT .90 threshold, which was adopted for this study.^60^ The relatively high HTMT value for PRIM and DIAL can be attributed to a possible conceptual overlap between these variables. Users engaging with the system after receiving feedback to achieve their primary goals may lead to interdependent responses. Moreover, when two constructs exhibit a strong yet not perfect correlation with values nearing 1.0, it is unlikely that the criterion will indicate discriminant validity, particularly with high loadings.^59^

**Table 2.** Discriminant validity using HTMT.

​	CONT	CRED	DIAL	EFFE	EFFO	PEPE	PRIM	TRAN	UNOB
CRED	0.547	​	​	​	​	​	​	​	​
DIAL	0.639	0.818	​	​	​	​	​	​	​
EFFE	0.846	0.578	0.657	​	​	​	​	​	​
EFFO	0.624	0.598	0.653	0.625	​	​	​	​	​
PEPE	0.731	0.751	0.832	0.794	0.688	​	​	​	​
PRIM	0.651	0.805	0.900	0.737	0.598	0.828	​	​	​
TRAN	0.590	0.692	0.686	0.693	0.690	0.766	0.749	​	​
UNOB	0.710	0.652	0.840	0.723	0.786	0.793	0.779	0.691	​
WeightCat	0.211	0.140	0.203	0.379	0.248	0.236	0.249	0.201	0.248

### Assessment of the structural model and hypotheses

The structure of the research model was tested by examining collinearity, the path coefficients, and explanatory and predictive power.^51^ On assessing the model for multicollinearity through the variance inflation factor (VIF), all values were below the recommended threshold (<5) as shown in Table 3, indicating no collinearity issues in our model.^51^ The significance of the path coefficients was determined by performing bootstrapping with 5000 samples (parallel processing, two-tailed percentile). All hypothesized relationships were significant. Consequently, the hypotheses regarding the relationships among the constructs, as well as the model’s structure, are deemed valid. Furthermore, effect sizes (f^2^) were used to determine the impact of path coefficients, using the guideline proposed by Cohen,^61^which can be small (0.02), medium (0.15), or large (0.35).^61^ It also depicts whether observed relationships are meaningful. Strong effects were observed for the relationship between PRIM → DIAL (f^2^ = 1.346) and CRED → TRAN (f^2^ = 0.730), indicating a strong impact, while small to medium effect sizes were observed for the rest of the paths (Table 3).

**Table 3.** Variance Inflation Factor (VIF), effect size, and hypothesis test.

​	VIF	Effect size (f^2^)	Hypothesis support
PRIM → DIAL	1.000	1.346	Yes
PRIM → PEPE	2.437	0.145	Yes
PRIM → UNOB	2.000	0.171	Yes
DIAL → UNOB	2.346	0.140	Yes
UNOB → PEPE	2.179	0.134	Yes
CRED → TRAN	1.000	0.730	Yes
TRAN → PEPE	2.012	0.090	Yes
PEPE → EFFE	1.644	0.274	Yes
PEPE → EFFO	1.000	0.575	Yes
PEPE→ CONT	1.575	0.258	Yes
EFFO → CONT	1.575	0.062	Yes
CONT → EFFE	1.644	0.374	Yes
EFFE → WeightCat	1.000	0.145	Yes

**Figure 3.**
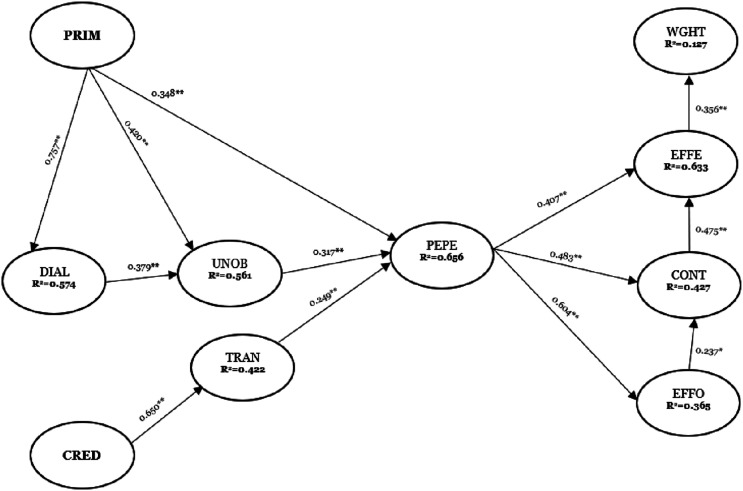
PLS-SEM results. *Note.* * p < .05; ** p ≤ .001.

Figure 3, shows that the highest variance explained in this model, using the coefficient of determination (R^2^), is derived from persuasive software features (PRIM) and characteristics (UNOB, TRAN), which collectively explained 65.6% of the variance in PEPE. PRIM and CRED accounted for 57.4% and 42.2% of the variance in DIAL and TRAN, respectively. Both PRIM and DIAL contributed to 56.1% of the variance in UNOB. PEPE explained 36.5% of the EFFO construct, while both PEPE and EFFO explained 42.7% of the CONT. Lastly, PEPE and CONT explained the second-highest variance, 63.3%, in EFFE. Overall, our model explains 12.7% of the variance observed in our outcome variable weight change.

In the model, some constructs mediated the relationships among other variables. The mediation can be either full or partial, depending on whether the existing direct relationship is significant or not.^62^ If a direct relationship is insignificant, the construct can be considered to fully mediate the relationship; otherwise, it is a partial mediator. Furthermore, partial mediation can be described as either complementary or competitive, depending on the sign of the path coefficient.^62^ To evaluate the four mediating relationships between constructs (H2b, H3b, H7b, H8b), we used the method outlined by Zhao et al.,^63^ by bootstrapping using 5000 subsamples and 95% bias-corrected confidence intervals. When comparing the direct and indirect effects, the results confirmed a partial and complementary mediation between the hypothesized relationships, as shown in Table 4.

**Table 4.** Mediation analysis (CI: 95% Confidence Interval).

​	Direct effect CI	p-value	Indirect effect CI	p-value
PRIM→ PEPE	[0.115, 0.329]	<0.001	[0.058, 0.248] via UNOB	0.007
PRIM→UNOB	[0.115, 0.329]	<0.001	[0.133, 0.430] via DIAL	<0.001
PEPE→CONT	[0.033, 0.280]	0.025	[0.033, 0.280] via EFFO	0.025
PEPE→EFFE	[0.212, 0.396]	<0.001	[0.128, 0.345] via CONT	<0.001

To check the generalizability of the findings, we performed the PLS_predict_ procedure and analyzed the root mean square error (RMSE).^51^ Results in Table 5 show that all PLS-SEM Q^2^ values exceed zero, meeting the requirement. Additionally, comparing the PLS-SEM RMSE values with the linear regression (LM) benchmark,^64^ shows that most indicators (13/21) have lower RMSE values than the LM benchmark, indicating medium predictive power.

**Table 5.** PLSpredict out-of-sample prediction (K = 10).

Items	Q^2^predict	PLS-SEM RMSE	LM RMSE
CONT2	0.180	1.363	1.397
CONT3	0.295	1.316	1.342
CONT4	0.151	1.418	1.474
PRA	0.594	1.039	1.019
REM	0.279	1.350	1.297
EFFE1	0.387	1.125	1.108
EFFE2	0.307	1.204	1.207
EFFE3	0.280	1.274	1.253
EFFO1	0.265	1.240	1.283
EFFO2	0.264	1.111	1.153
EFFO3	0.096	1.533	1.526
PEPE1	0.594	0.920	0.875
PEPE3	0.449	1.248	1.304
PEPE4	0.296	1.215	1.252
TRAN1	0.365	1.041	1.008
TRAN2	0.286	1.139	1.151
TRAN3	0.288	1.130	1.120
UNOB1	0.479	1.230	1.275
UNOB2	0.475	1.211	1.265
UNOB3	0.187	1.736	1.791
WeightCat	0.044	1.092	1.110

*Note.* RMSE = Root Mean Squared Error, LM = Linear regression model.

We incorporated age as a continuous moderator of the relationship between EFFE and weight change to examine its effect on the model. Upon conducting the analysis and applying the bootstrapping procedure, the findings revealed that age significantly affects the association between EFFE and weight change, increasing the model’s explanatory power (R^2^ = 16.3%). Additionally, there is a slight increase in the effect size (f^2^ = 0.151 from 0.145) in this relationship (*β *= -0.197, p =0.013, f^2^ = 0.038).

## Discussion

We investigated the impact of persuasive design principles (PRIM, DIAL, CRED, UNOB, TRAN) on perceived persuasiveness, BCSS acceptance factors (perceived effort, continuance intention, perceived effectiveness), and overall weight loss outcomes. Using a subset of data collected from users of a weight management intervention during an RCT, we developed and evaluated a PLS-SEM model. All hypothesized relationships were significant. Overall, the proposed model explained 12.7% of the variance in the weight change outcome variable and had medium predictive power.

PRIM and DIAL (through unobtrusiveness), as well as CRED (via transparency), have a significant and positive effect on perceived persuasiveness, jointly explaining 65.6% of the variance. This highlights the role of persuasive features within these categories in shaping users’ perceptions of persuasiveness in weight management interventions, particularly the importance of unobtrusiveness and transparency as underlying mechanisms.^43,65^ Moreover, PRIM had a strong effect on DIAL (f^2^ = 1.346), suggesting that when users perceive the system as useful for achieving their intended goals, they are more open to feedback through DIAL features such as reminders and praise. Likewise, CRED had a strong effect on transparency (f^2^ = 0.730), indicating that when users perceive a system as credible/reliable, they perceive greater transparency. Our findings highlight that credibility reinforces transparency in a system, which in turn leads to perceived persuasiveness.

The findings indicated that, although statistically significant, several hypothesized relationships (i.e., PRIM → PEPE, UNOB → PEPE, TRAN → PEPE, DIAL → UNOB, EFFO → CONT, and EFFE → Weight category) had small effect sizes (f^2^ < 0.15). However, the significance of these associations suggests that even small effects from design features (DIAL and PRIM), along with characteristics of unobtrusiveness and transparency, collectively influence users’ perceptions of persuasiveness. Therefore, slight improvements in these elements could yield meaningful behavior and health outcomes,^7^ highlighting the practical relevance of our findings.

Dialogue support partially mediated the relationship between PRIM and UNOB (H2b), suggesting that unobtrusive system design influences perceptions of primary task support when mediated through effective feedback mechanisms such as reminders and suggestions. Similarly, UNOB partially mediated (H3b) the relationship between PRIM and PEPE, indicating that when a system is perceived as unobtrusive, it can enhance the user’s interaction with the system as the user pursues desired behavioral goals. Minimizing distractions allows users to focus on their tasks, creating a smooth, enjoyable experience. Also, an unobtrusive system fits seamlessly into the user’s life. Such systems are typically aware of the user’s context and adapt to the user’s needs over time, without demanding unnecessary attention.^66^ Along the same lines, findings of a systematic review indicate that unobtrusiveness influenced users’ willingness to use mHealth apps.^39^

Moreover, perceived effort (H7b) partially mediated and complemented the relationship between perceived persuasiveness and continuance intention. In other words, when users find a system persuasive, they perceive it as requiring less effort, which increases their intention to continue using it. This is consistent with the findings of Beldad and Hegner,^21^ who studied a similar behavior change intervention related to fitness. In the same way, continuance intention partially mediated the relationship between PEPE and perceived effectiveness. When users perceive a system as persuasive, they are more likely to continue using it, thereby increasing their perceptions of its effectiveness. The continued use of a system may render it effective for that purpose.

### Theoretical and practical implications

Our findings contribute to the theoretical understanding of persuasive systems design and its practical application to digital health interventions. The proposed model explained 12.7% of the variance in the outcome variable, weight change. While this R^2^ value is relatively modest, this finding aligns with prior research on persuasive systems design and health outcomes, such as weight loss.^65,67^ For example, a recent study on PSD and goal setting reported a 15.3% variance explaining body mass index reduction as an outcome variable,^65^ thus supporting the role of behavior change theories in facilitating health outcomes.

Integrating the PSD framework and TAM provides an explanation of how persuasive systems design translates into user acceptance and continued use of digital health applications. While earlier studies have explored PSD and its impact on perceived effectiveness and user continuance intention,^24,28^ recent studies have begun incorporating weight-related outcomes.^65,67^ Building on this emerging work, this study advances the literature by incorporating an observable outcome, weight loss, alongside PSD and technology acceptance constructs. This integration highlights the potential of persuasive systems design to increase the effectiveness of weight management interventions, consistent with prior evidence on persuasive design and behavior change.^15^ The significant partial mediating role of CONT in the relationship between PEPE and EFFE extends research on PSD, technology acceptance, and behavior change. The perceived effectiveness of a system is directly influenced by how convincing users find it and indirectly by the ongoing intention to continue using it. This highlights that predicting system effectiveness may be based on users’ initial perceptions of persuasiveness and ongoing engagement.

Furthermore, this study’s findings provide insights into system features and characteristics that influence persuasiveness, thereby enhancing ease of use, usefulness, and system effectiveness. For instance, a weight management app designed with PRIM features (such as self-monitoring and tailoring) and unobtrusive, timely feedback through reminders, praise, and suggestions can increase users’ perceptions of its persuasiveness. These positive perceptions, in turn, decrease the perceived effort needed to interact with the app, positively impact its perceived effectiveness, and influence the intention to continue using it, which is a key determinant of technology adoption.

In practice, the combined impacts of system features (PRIM, DIAL, CRED) and postulates (unobtrusiveness and transparency) influence how users perceive a system as persuasive. This indicates that features under these categories work together to influence a user’s behavior. Research has highlighted that these features are often implemented in combination to create a synergistic effect within the system.^10^ Consequently, designers should explore the best combinations of these features that increase a user’s perceptions of persuasiveness. For instance, in the intervention used in this study, PRIM’s self-monitoring feature was closely implemented with reminders (DIAL), unobtrusively delivering them only when needed. In addition, credibility-related features, such as clear communication of system purpose and data use, support user trust and may further strengthen the system’s perceived persuasiveness.

Our findings highlight the importance of unobtrusive system design, particularly dialogue-enhancing features, in increasing a system’s perceived persuasiveness. While DIAL features (e.g., reminders, suggestions) have been implemented in similar interventions to boost motivation and encourage system engagement,^37^ we argue that such features must be delivered without distracting users to increase their effectiveness. For instance, a praise message delivered within the app (in-app) rather than a pop-up, after users log their physical activity or weight, supports their behavioral goals without disruption. Moreover, allowing users to opt in to receive these notifications or to customize the timing and frequency of prompts makes them less obtrusive. Moreover, introducing age as a moderator between perceived effectiveness and weight change increased the model’s explanatory power (from R^2^ = 12.7% to 16.3%) and effect size from small (f^2^ = 0.145) to medium (f^2^ = 0.151). The significant negative path coefficient (β = -0.197, p = 0.013) suggests that a system’s perceived effectiveness in influencing weight loss is more impactful among younger participants than older users. This finding highlights the importance of considering age when designing interventions, as perceptions may vary with age. Additionally, it highlights the potential influence of other factors, including psychological aspects such as a user’s level of motivation, cognitive consistency, level of physical activity, and nutrition, which could affect behavior, as well as actual health outcomes like weight loss. Table 6 presents a summary of the practical design recommendations.

**Table 6.** Design implications for developing persuasive health interventions for weight loss.

Design aspect	Recommendation	Example
Feature combination (PRIM, DIAL, CRED)	Combine system features to create a synergistic effect on user persuasiveness	Integrate self-monitoring with reminders by linking reminder prompts to user activity and progress
Dialogue support	Use reminders and suggestions to boost motivation and encourage engagement	Send a reminder for missed user expected actions (i.e., missed task completion or weight log)
Unobtrusiveness	Deliver system features without distracting users to increase effectiveness	Provide in-app praise messages, text, or email instead of pop-ups
		Provide structured delivery of DIAL messages, i.e., pre-defined schedule (day and time)
​	​	Enable users to opt in to receive notifications or customize the timing and frequency
System credibility & Transparency	Strengthen user trust through transparent and credible system design	Provide evidence-based content, clearly communicate system goals and key strategies as part of the onboarding process, and disclose how data will be used
Age	Consider age-related differences in perceived effectiveness when designing interventions	Adapt intervention design (i.e., engagement strategies) to account for differences across age groups

### Limitations and future research

While the results provide valuable implications, the study has some limitations. Our sample had a higher representation of females (87.3%) than males, which may limit the generalizability of the findings. However, this sample distribution is not surprising, as studies have shown that women tend to be more concerned about their health and are therefore more inclined to address it.^68^ Similarly, a recent review suggests that the participants in weight interventions are mostly women.^69^ Moreover, our sample size did not meet the required threshold (n = 69) for subgroup analysis.^52^ Another potential limitation is the risk of common method variance (CMV), as most perceptual constructs were measured using a single self-report questionnaire administered at one time point. However, the primary outcome variable, weight change, was measured objectively by healthcare professionals, thereby reducing the likelihood that CMV would affect the relationship between predictors and the outcome. While CMV may still influence relationships among perceptual constructs, future research could consider additional procedural or statistical approaches, such as temporal separation measures or post hoc diagnostics, to further assess its impact. Moreover, beyond age, the model does not control for other potential confounders such as baseline BMI, pre-intervention physical activity levels, or comorbidities. In a sample with BMI ranging from 30 to 40 kg/m^2^, these variables may account for part of the residual variance in weight outcomes and could contribute to the relatively modest R^2^ of 12.7%.

Future research should aim for more balanced gender representation to enhance the robustness and generalizability of the findings. It should also consider including additional control variables to improve the model’s explanatory power. Furthermore, future research can expand the breadth and depth of knowledge of persuasive systems for behavior change and contribute to a more comprehensive understanding of the intricate interplay among variables, including user perceptions of system features and other relevant constructs such as social support. While this study adopts a quantitative approach, future research could incorporate qualitative methods to provide a more nuanced understanding of users’ experiences with these system features and their effects on behavior modification.

However, as the model assumes a unidirectional effect from perceived effectiveness to weight loss, we did not exclude the possibility of a bidirectional relationship. Future longitudinal studies are needed to clarify these reciprocal relationships. Notwithstanding these limitations, the model passed the relevant tests to validate the findings and contributes to explaining the system-related factors, perceived behavior, and their impact on actual health outcomes, such as weight loss.

## Conclusion

This study explored how persuasive design principles (PRIM, DIAL, CRED, UNOB, TRAN) affect perceived persuasiveness, perceived effort, intention to continue using the mobile intervention, perceived effectiveness, and overall weight change outcomes. The results show that primary task support, dialogue support (via unobtrusiveness), and credibility support jointly influence the perceived persuasiveness of a system and the intention to sustain usage. Moreover, the importance of system characteristics such as unobtrusiveness, transparency, and ease of use (effort) was highlighted. Our proposed model explained 12.7% of the variance in weight change, with medium predictive power, extending prior research on persuasive systems design and continuance intention. These findings suggest that optimizing these features can effectively help users achieve their behavioral goals, contributing to the understanding persuasive systems design and technology acceptance of digital health interventions. The study’s outcomes provide insights for researchers, designers, and developers of weight management and health behavior change apps.

## Supplemental material

Supplemental material - Evaluating the Influence of Persuasive Systems Design on Continuance Intention, Perceived Effectiveness, and Weight Loss Supplemental material for Evaluating the Influence of Persuasive Systems Design on Continuance Intention, Perceived Effectiveness, and Weight Loss by Sharon Nabwire, Eunice Agyei, Heta Merikallio, Markku J. Savolainen, Janne Hukkanen, and Harri Oinas-Kukkonen in Digital Health.

## Data Availability

The data supporting this study’s findings are used solely for research purposes and are not publicly available due to privacy and ethical restrictions. Reasonable data requests may be directed to the corresponding author.*

## Appendix

### Appendix A: Item loading for the various constructs

**Table:**

Construct	Concept origin	Item/Description	Loading
Primary task support (PRIM)	Oinas-Kukkonen & Harjumaa^7^; Klein et al.^35^; Fogg & Nass^34^	RED: The system provides information that helps me to gradually reach my goals.	0.949
		SMO: The system provides information that helps me keep track of my progress.	0.907
		TAI: The system provides information that suits people like me.	0.889
Dialogue support (DIAL)	Oinas-Kukkonen & Harjumaa^7^	REM: The system reminds me about my personal goals.	0.866
		PRA: The system encourages me.	0.921
Credibility support	Oinas-Kukkonen & Harjumaa^7^	EXP1: Overall, I consider the system accurate.	0.971
		EXP2: Overall, I consider the system professional.	0.952
		TRU1: Overall, I consider the system believable.	0.967
		TRU2: Overall, I consider the system trustworthy.	0.954
Unobtrusiveness (UNOB)	Oinas-Kukkonen & Harjumaa^7^; Lehto et al.^44^	UNOB1: Using the system fits into my daily life.	0.958
		UNOB2: Using the system is convenient for me.	0.945
		UNOB3: Finding the time to use the system is not a problem for me.	0.765
Transparency (TRAN)	Oinas-Kukkonen & Harjumaa^7^	TRAN1: The message conveyed through the system is clear.	0.883
		TRAN2: I can recognize the actual source of the coaching given by the system.	0.924
		TRAN3: The basis for the provided coaching is explained thoroughly.	0.918
Perceived Effort (EFFO)	Davis^20^; Venkatesh et al.^33^	EFFO1: Using the system does not require a lot of effort from me.	0.910
		EFFO2: Using the system is straightforward for me.	0.890
		EFFO3: (Negation) Using the system is laborious.	0.795
Perceived Effectiveness (EFFE)	Venkatesh et al.^33,50^; Lehto & Oinas-Kukkonen^24^	EFFE1: My chances of reaching my goal improve by using the system.	0.927
		EFFE2: In my opinion, using the system has an effect on my result.	0.930
		EFFE3: (Negation) In my opinion, the system has no effect on my result.	0.855
Perceived persuasiveness (PEPE)	Oinas-Kukkonen^6^; Lehto et al.^46^	PEPE1: In my view, the system is convincing.	0.870
		PEPE2: In my view, the system is intriguing.	0.937
		PEPE3: The system is appealing.	0.875
Continuance intention (CONT)	Bhattacherjee^48^; de Guinea & Markus,^49^	CONT1: I will be using a similar system in the future.	0.800
		CONT2: (Negation) I am considering discontinuing using the system.	0.882
		CONT3: (Negation) I am not going to use this kind of a system from now on.	0.847
Weight change (WGHT)	​	1: x > 5% weight loss	1.000
		2: 2% < x ≤ 5% weight loss	
		3: 0% < x ≤ 2% weight loss	
		4: x ≤ 0% weight loss	

*Note.* The negations were rescaled to be positive by reversing the 1-7 scale so that 1 = strongly agree and 7 = strongly disagree, ensuring consistent interpretation throughout items.

### Appendix B: Participant characteristics

**Table:**

​	​	n=181
Characteristics	Item	Frequency (%)
Sex	Female	158 (87.29)
	Male	23 (12.71)
Age	18-29	5 (2.76)
	30-39	32 (17.68)
	40-49	67 (37.02)
	50-59	59 (32.60)
	60-69	18 (9.94)
Weight Change Categories	x > 5% weight loss (Cat 1)	36 (19.89)
	2% < x ≤ 5% weight loss (Cat 2)	43 (23.76)
	0% < x ≤ 2% weight loss (Cat 3)	46 (25.41)
	x ≤ 0% weight loss (Cat 4)	56 (30.94)
Marital Status	Widowed	1 (0.55)
	Divorced	20 (11.05)
	Cohabitation	27 (14.92)
	Married	114 (62.98)
	Unmarried	19 (10.50)
Professional Training	Professional course (at least 4 months)	2 (1.10)
	Other employment course (at least 4 months)	1 (0.55)
	Vocational school	25 (13.81)
	University of Applied Sciences	89 (49.17)
	University	44 (24.31)
	None of the earlier-mentioned	20 (11.05)

## References

[1] Blüher M . Obesity: global epidemiology and pathogenesis. Nat Rev Endocrinol, 15 2019; 288-298. 10.1038/s41574-019-0176-8 30814686

[2] Ng M Fleming T Robinson M , et al. Global, regional, and national prevalence of overweight and obesity in children and adults during 1980-2013: A systematic analysis for the Global Burden of Disease Study 2013. The Lancet 2014; 384: 766–781. 10.1016/S0140-6736(14)60460-8 PMC4624264 24880830

[3] Wadden TA Tronieri JS Butryn ML . Lifestyle modification approaches for the treatment of obesity in adults. American Psychologist 2020; 75: 235–251. 10.1037/amp0000517 32052997 PMC7027681

[4] Chiavarini M Giacchetta I Rosignoli P , et al. E-Health and M-Health in Obesity Management: A Systematic Review and Meta-Analysis of RCTs. Nutrients 2025; 17: 2200. 10.3390/nu17132200 40647304 PMC12251417

[5] Li S Zhou Y Tang Y , et al. Behavior Change Resources Used in Mobile App–Based Interventions Addressing Weight, Behavioral, and Metabolic Outcomes in Adults With Overweight and Obesity: Systematic Review and Meta-Analysis of Randomized Controlled Trials. JMIR Mhealth Uhealth 2025; 13: e63313. 10.2196/63313 40829125 PMC12392691

[6] Oinas-Kukkonen H . A foundation for the study of behavior change support systems. Pers Ubiquitous Comput 2013; 17: 1223–1235. 10.1007/s00779-012-0591-5

[7] Oinas-Kukkonen H Harjumaa M . Persuasive systems design: Key issues, process model, and system features. Communications of the Association for Information Systems 2009; 24: 485–500. 10.17705/1cais.02428

[8] Alhasani M Orji R . Promoting Stress Management among Students in Higher Education: Evaluating the Effectiveness of a Persuasive Time Management Mobile App. Int J Hum Comput Interact 2025; 41: 219–241. 10.1080/10447318.2023.2297330

[9] Oyibo K Adaji I Orji R , et al. Perceived persuasive effect of behavior model design in fitness apps. In: UMAP 2018 - Proceedings of the 26th Conference on User Modeling, Adaptation and Personalization. Association for Computing Machinery, Inc, 2018, pp. 219–228. 10.1145/3209219.3209240

[10] de Oliveira SCR Oinas-Kukkonen H . Evidence Levels for Persuasive Software Features in Digital Health Interventions: Insights from a Scoping Review Regarding T2DM. ICIS 2024 Proceedings, 2024; https://aisel.aisnet.org/icis2024/ishealthcare/ishealthcare/15

[11] Michie S van Stralen MM West R . The behaviour change wheel: A new method for characterising and designing behaviour change interventions. Implementation Science 2011; 6: 42. 10.1186/1748-5908-6-42 21513547 PMC3096582

[12] McLean A . mHealth Apps as Effective Persuasive Health Technology: Contextualizing the “Necessary” Functionalities. JMIR Nurs 2020; 3: e19302. 10.2196/19302 34345788 PMC8279448

[13] Fogg BJ . A behavior model for persuasive design. In: ACM International Conference Proceeding Series, 2009; 1-7. 10.1145/1541948.1541999

[14] Asbjørnsen RA Wentzel J Smedsrød ML , et al. Identifying Persuasive Design Principles and Behavior Change Techniques Supporting End User Values and Needs in eHealth Interventions for Long-Term Weight Loss Maintenance: Qualitative Study. J Med Internet Res 2020; 22: e22598. 10.2196/22598 33252347 PMC7735908

[15] Markkanen JO Oikarinen N Savolainen MJ , et al. Mobile health behaviour change support system as independent treatment tool for obesity: a randomized controlled trial. Int J Obes 2023; 48: 376. 10.1038/s41366-023-01426-x PMC10896717 38062218

[16] Lehto T . Designing Persuasive Health Behavior Change Interventions. In: Critical Issues for the Development of Sustainable E-health Solutions. Springer US, 2012, pp. 163–181, 10.1007/978-1-4614-1536-7_11

[17] Sittig S McGowan A Iyengar S . Extensive Review of Persuasive System Design Categories and Principles: Behavioral Obesity Interventions. Journal of Medical Systems 2020; 44: 128. 10.1007/s10916-020-01591-w. 32500161

[18] Webb TL Joseph J Yardley L , et al. Using the Internet to promote health behavior change: A systematic review and meta-analysis of the impact of theoretical basis, use of behavior change techniques, and mode of delivery on efficacy. J Med Internet Res 2010; 12: e4. 10.2196/jmir.1376 20164043 PMC2836773

[19] Johnson BT Scott-Sheldon LAJ Carey MP . Meta-Synthesis of Health Behavior Change Meta-Analyses. Am J Public Health 2010; 100: 2193–2198. 10.2105/ajph.2008.155200 20167901 PMC2951968

[20] Davis FD . Perceived Usefulness, Perceived Ease of Use and User Acceptance of Information Technology. MS Quarterly, 1989; 13: 319-340. 10.2307/249008

[21] Beldad AD Hegner SM . Expanding the Technology Acceptance Model with the Inclusion of Trust, Social Influence, and Health Valuation to Determine the Predictors of German Users’ Willingness to Continue using a Fitness App: A Structural Equation Modeling Approach. Int J Hum Comput Interact 2018; 34: 882–893. 10.1080/10447318.2017.1403220

[22] Bettiga D Lamberti L Lettieri E . Individuals’ adoption of smart technologies for preventive health care: a structural equation modeling approach. Health Care Manag Sci 2020; 23: 203–214. 10.1007/s10729-019-09468-2 30684067

[23] Miao R Wu Q Wang Z , et al. Factors that influence users’ adoption intention of mobile health: a structural equation modeling approach. Int J Prod Res 2017; 55: 5801–5815. 10.1080/00207543.2017.1336681

[24] Lehto T Oinas-Kukkonen H . Explaining and predicting perceived effectiveness and use continuance intention of a behaviour change support system for weight loss. Behaviour and Information Technology 2015; 34: 176–189. 10.1080/0144929x.2013.866162

[25] Oduor M Oinas-Kukkonen H . Commitment Devices as Behavior Change Support Systems: A Study of Users’ Perceived Competence and Continuance Intention. In: International Conference on Persuasive Technology. Lecture Notes in Computer Science(), vol 10171. Springer, Cham., 2017, pp. 201–213. 10.1007/978-3-319-55134-0_16

[26] Shoneye CL Mullan B Begley A , et al. Design and Development of a Digital Weight Management Intervention (ToDAy): Qualitative Study. JMIR Mhealth Uhealth 2020; 8: e17919. 10.2196/17919 32641284 PMC7511863

[27] Karppinen P Oinas-Kukkonen H Alahäivälä T , et al. Persuasive user experiences of a health Behavior Change Support System: A 12-month study for prevention of metabolic syndrome. Int J Med Inform 2016; 96: 51–61. 10.1016/j.ijmedinf.2016.02.005 26992482

[28] Wiafe I Koranteng FN Kastriku FA , et al. Assessing the impact of persuasive features on user’s intention to continuous use: the case of academic social networking sites. Behaviour and Information Technology 2020; 41: 712–730. 10.1080/0144929x.2020.1832146

[29] Räisänen T Lehto T Oinas-Kukkonen H . Practical Findings from Applying the PSD Model for Evaluating Software Design Specifications. In: Persuasive Technology: 5th International Conference, PERSUASIVE 2010. Springer, 2010, pp. 185–192. 10.1007/978-3-642-13226-1_19

[30] Davis FD . User acceptance of information technology: system characteristics, user perceptions and behavioral impacts. Int J Man Mach Stud 1993; 38: 475–487. 10.1006/imms.1993.1022

[31] Radner R Rothschild M . On the allocation of effort. J Econ Theory 1975; 10: 358–376. 10.1016/0022-0531(75)90006-x

[32] Venkatesh V Brown SA . A Longitudinal Investigation of Personal Computers in Homes: Adoption Determinants and Emerging Challenges. MIS Quarterly 2001; 25: 71–102. 10.2307/3250959

[33] Venkatesh V Smith RH Morris MG , et al. User acceptance of information technology: Towards a unified view. MIS quarterly 2003: 425–478. 10.2307/30036540

[34] Fogg BJ Nass C . Silicon sycophants: the effects of computers that flatter. Int J Hum Comput Stud 1997; 46: 551–561. 10.1006/ijhc.1996.0104

[35] Klein J Moon Y Picard RW . This computer responds to user frustration. Interact Comput 2002; 14: 119–140. 10.1016/s0953-5438(01)00053-4

[36] Derrick DC Jenkins JL Nunamaker JF . Design Principles for Special Purpose, Embodied, Conversational Intelligence with Environmental Sensors (SPECIES) Agents. In: AIS Transactions on Human-Computer Interaction, 2011; 62-81. https://aisel.aisnet.org/thci/vol3/iss2/2

[37] Nabwire S de Oliveira RSC Merikallio H , et al. Evaluating Persuasive Reminders and Suggestions in a Weight Management mHealth Intervention. In: 59th Hawaii International Conference on System Sciences, 2026, pp. 3777–3786. 10.24251/hicss.2026.452

[38] Halttu K Oinas-Kukkonen H . Persuading to Reflect: Role of Reflection and Insight in Persuasive Systems Design for Physical Health. Hum Comput Interact 2017; 32: 381–412. 10.1080/07370024.2017.1283227

[39] Niezen MGH . Unobtrusiveness in mHealth Design and Use: A Systematic Literature Study. In: Under Observation: The Interplay Between eHealth and Surveillance. Law, Governance and Technology Series(), vol 35. Springer, Cham, 2017, pp. 9–29. 10.1007/978-3-319-48342-9_2

[40] Fogg BJ Tseng H . The elements of computer credibility. In: Proceedings of the SIGCHI conference on Human factors in computing systems the CHI is the limit - CHI ’99. ACM Press, 1999, pp. 80–87. 10.1145/302979.303001

[41] Shin D . User Perceptions of Algorithmic Decisions in the Personalized AI System:Perceptual Evaluation of Fairness, Accountability, Transparency, and Explainability. J Broadcast Electron Media 2020; 64: 541–565. 10.1080/08838151.2020.1843357

[42] Gamerdinger A Just SN Lantz PMV . Healthy transparency: Dynamic interrelations between credibility, transparency, and trust in the context of Danish public authorities’ COVID-19 communication. Social Sciences & Humanities Open 2023; 8: 100688. 10.1016/j.ssaho.2023.100688

[43] Nabwire S de Oliveira R Merikallio H , et al. Enhancing the perceived effectiveness of health behaviour change support systems through transparency in persuasive systems design. Behaviour & Information Technology 2026; 10.1080/0144929X.2026.2638915

[44] Lehto T Oinas-Kukkonen H Drozd F . Factors affecting perceived persuasiveness of a Behavior Change Support System. In: International Conference on Information Systems, 2012. https://aisel.aisnet.org/icis2012/proceedings/HumanBehavior/18 *ICIS 2012* .

[45] Beerlage-de Jong N Kip H Kelders SM . Evaluation of the Perceived Persuasiveness Questionnaire: User-Centered Card-Sort Study. J Med Internet Res 2020; 22: e20404. 10.2196/20404 33095173 PMC7647815

[46] Drozd F Lehto T Oinas-Kukkonen H . Exploring Perceived Persuasiveness of a Behavior Change Support System: A Structural Model. In: 7th International Conference, PERSUASIVE 2012. Linköping. Springer, 2012, pp. 157–168, 10.1007/978-3-642-31037-9_14

[47] Shevchuk N Degirmenci K Oinas-Kukkonen H . Adoption of Gamified Persuasive Systems to Encourage Sustainable Behaviors: Interplay between Perceived Persuasiveness and Cognitive Absorption. ICIS 2019 Proceedings. Association for Information System, 2019, https://aisel.aisnet.org/icis2019/behavior_is/behavior_is/3/

[48] Bhattacherjee A . Understanding Information Systems Continuance: An Expectation-Confirmation Model. l. MIS Quarterly, 2001; 351-370. 10.2307/3250921

[49] de Guinea AO Markus ML . Why Break the Habit of a Lifetime? Rethinking the Roles of Intention, Habit, and Emotion in Continuing Information Technology use. MIS Quarterly 2009; 33: 433–444. 10.2307/20650303

[50] Venkatesh T Thong JYL Xu X . Consumer Acceptance and Use of Information Technology: Extending the Unified Theory of Acceptance and Use of Technology. MIS Quarterly 2012; 36: 157–178. 10.2307/41410412

[51] Hair JF Hult GTM Ringle CM , et al. A Primer on Partial Least Squares Structural Equation Modeling (PLS-SEM). 3rd ed.: Sage, 2022.

[52] Kock N Hadaya P . Minimum sample size estimation in PLS-SEM: The inverse square root and gamma-exponential methods. Information Systems Journal 2018; 28: 227–261. 10.1111/isj.12131

[53] Barclay DW Higgins C Thompson R . The partial least squares (PLS) approach to causal modeling: Personal computer adoption and use as an illustration. Technology Studies 1995; 2: 285–309.

[54] Eight‐year weight losses with an intensive lifestyle intervention: The look AHEAD study. Obesity 2014; 22: 5–13. 10.1002/oby.20662 24307184 PMC3904491

[55] Hair JF Hult GTM Ringle CM , et al. An Introduction to Structural Equation Modeling. In: Partial Least Squares Structural Equation Modeling (PLS-SEM) Using R. Springer, 2021, pp. 1–29.

[56] Lowry PB Gaskin J . Partial Least Squares (PLS) Structural Equation Modeling (SEM) for Building and Testing Behavioral Causal Theory: When to Choose It and How to Use It. IEEE Trans Prof Commun 2014; 57: 123–146. 10.1109/tpc.2014.2312452

[57] Foldnes N Grønneberg S . The sensitivity of structural equation modeling with ordinal data to underlying non-normality and observed distributional forms. Psychol Methods 2022; 27: 541–567. 10.1037/met0000385 33793270

[58] Ringle CM Wende S Becker J-M . SmartPLS 4. https://www.smartpls.com

[59] Henseler J Ringle CM Sarstedt M . A new criterion for assessing discriminant validity in variance-based structural equation modeling. J Acad Mark Sci 2015; 43: 115–135. 10.1007/s11747-014-0403-8

[60] Gold AH Malhotra A Segars AH . Knowledge Management: An Organizational Capabilities Perspective. Journal of Management Information Systems 2001; 18: 185–214. 10.1080/07421222.2001.11045669

[61] Cohen J . Routledge, 2013. 10.4324/9780203771587, Statistical Power Analysis for the Behavioral Sciences.

[62] Nitzl C Roldan JL Cepeda G . Mediation analysis in partial least squares path modeling. Industrial Management & Data Systems 2016; 116: 1849–1864. 10.1108/imds-07-2015-0302

[63] Zhao X Lynch JG Chen Q . Reconsidering Baron and Kenny: Myths and truths about mediation analysis. Journal of Consumer Research 2010; 37: 197–206. 10.1086/651257

[64] Shmueli G Sarstedt M Hair JF , et al. Predictive model assessment in PLS-SEM: guidelines for using PLSpredict. Eur J Mark 2019; 53: 2322–2347. 10.1108/ejm-02-2019-0189

[65] de Oliveira RSC Nabwire S Merikallio H , et al. Behind the software: The impact of Unobtrusiveness, Goal Setting and persuasive features on BMI. Int J Med Inform 2025; 196: 105795. 10.1016/j.ijmedinf.2025.105795 39862566

[66] Bakker S Niemantsverdriet K . The interaction-attention continuum: considering various levels of human attention in interaction design. International Journal of Design 2016; 10: 1–14.

[67] Nabwire S de Oliveira SR Merikallio H , et al. Cognitive Consistency as a Facilitator of Perceived Persuasiveness and Continuance Intention in Digital Weight Management. In: ICIS 2025 Proceedings. Association for Information Systems, 2025. https://aisel.aisnet.org/icis2025

[68] Thompson AE Anisimowicz Y Miedema B , et al. The influence of gender and other patient characteristics on health care-seeking behaviour: a QUALICOPC study. BMC Fam Pract 2016; 17: 38. 10.1186/s12875-016-0440-0 27036116 PMC4815064

[69] Hallock R Ufholz K Patel N . Self-Monitoring of Weight as a Weight Loss Strategy: A Systematic Review. Curr Cardiovasc Risk Rep 2024; 18: 163–172. 10.1007/s12170-024-00746-5

