# Supplementary material for: Evaluating the influence of persuasive systems design on continuance intention, perceived effectiveness, and weight loss
Source: Digit Health. 2026 Jun 16;12:20552076261461365. doi: 10.1177/20552076261461365 (PMC13272979; doi:10.1177/20552076261461365)
Supplement: Supplemental material - Evaluating the Influence of Persuasive Systems Design on Continuance Intention, Perceived Effectiveness, and Weight Loss [file sj-pdf-1-dhj-10.1177_20552076261461365.pdf]

Supplementary Material: Survey items used in this study

Target: To determine perceptions of using the information system and its parts, and acceptance of the system

Please indicate how much you agree with the following statements about the system on a scale of 1 to 7, with 1 representing strongly agree and 7 representing strongly disagree.

1. The system provides information that helps me to gradually reach my goals.
   The system provides information that helps me keep track of my progress.
   The system provides information that suits people like me.

2. The system reminds me about my personal goals
   The system encourages me.

3. Overall, I consider the system accurate
   Overall, I consider the system professional.
   Overall, I consider the system believable
   Overall, I consider the system trustworthy.

4. Using the system fits into my daily life
   Using the system is convenient for me
   Finding the time to use the system is not a problem for me

5. The message conveyed through the system is clear
   I can recognize the actual source of the coaching given by the system
   The basis for the provided coaching is explained thoroughly

6. Using the system does not require a lot of effort from me
   Using the system is straightforward for me
   Using the system is laborious

7. My chances of reaching my goal improve by using the system
   In my opinion, using the system has an effect on my result
   In my opinion, the system has no effect on my result

8. In my view, the system is convincing.
   In my view, the system is intriguing
   The system is appealing.

9. I will be using a similar system in the future
   I am considering discontinuing using the system.
   I am not going to use this kind of a system from now on.
